# Modeling of abnormal grain growth in (111) oriented and nanotwinned copper

**DOI:** 10.1038/s41598-021-99992-5

**Published:** 2021-10-14

**Authors:** A. M. Gusak, Kuan-Ju Chen, K. N. Tu, Chih Chen

**Affiliations:** 1 Department of Physics, Cherkasy National University, Cherkasy, Ukraine; 2 Department of Materials Science and Engineering, University of California at Los Angeles, Los Angeles, CA USA; 3 International College of Semiconductor Technology, National Chiao Tung University, Hsinchu, Taiwan, ROC; 4 Department of Materials Science and Engineering, National Chiao Tung University, Hsinchu, Taiwan, ROC; 5 Departmant of Materials Science and Engineering, National Yang Ming Chiao Tung University, Hsinchu, Taiwan, ROC

**Keywords:** Electronic devices, Chemical physics, Surfaces, interfaces and thin films, Atomistic models

## Abstract

Uni-modal, not bi-modal, of abnormal grain growth has been observed in (111) oriented and nano-twinned Cu films. Because of the highly anisotropic microstructure, our kinetic analysis and calculation showed that it is the mobility which dominates the uni-modal growth, in which the lateral growth rate can be two orders of magnitude higher than the vertical growth rate. As a consequence, the abnormal grain growth has been converted from bi-modal to uni-modal.

## Introduction

Copper (Cu) is considered as the most widely used interconnect in microelectronic devices^1^. It has been employed to replace aluminum thanks to its great electrical conductivity. As the physical dimension of interconnects is required to significantly scale down to sub-micrometer for the current advanced packaging, the reliability related to thermomigration and electromigration (EM) becomes critical concerns^2,3^. It has been reported that, by introducing nanotwinned Cu (nt-Cu), the reliability issues could be greatly suppressed^4^. Additionally, nt-Cu was demonstrated to possess superior mechanical properties^5–8^. Thus, it has potential for the future implementation in the microelectronic industry.

Abnormal grain growth occurs in polycrystalline Cu. It is abnormal because of the bi-modal distribution of grains, where a small number of very large grains whose diameter can be hundred times larger than the rest of the small grains in the matrix. We report here that when densely (111)-orientated nanotwins are introduced into Cu, abnormal grain growth occurs too^9–14^. However, the distribution of grains becomes uni-modal, not bi-modal, because all the grains become several hundred microns in diameter in a Cu thin film of thickness of a few microns. The critical role of the oriented nanotwins on affecting the uni-modal abnormal grain growth has been analyzed on the basis of anisotropic grain boundary mobility in the vertical vs. the lateral directions of the nanotwins.

We recall that the formation of bi-modal abnormal grains in Cu has been attributed to the balance between strain energy and surface energy^15,16^. It is due to the change of elastic modulus (and the corresponding decrease of the thermal stress energy) in transforming (111) to (200) orientation during thermal annealing^17^. When the energetic condition is right, those abnormally large grains can grow with the (200) orientation, because the (200) oriented grains have less strain energy.

Morphologically, the average grain size in a thin film is typically about the film thickness. Thus, in a uni-modal abnormal grain, when the lateral dimension becomes hundred times larger than the film thickness, it means that the lateral growth rate is hundred times faster than the vertical growth rate, so the growth is highly anisotropic. Why can it occur and what is the driving force and the kinetic processes behind the uni-modal abnormal growth require a systematic study. Our aim is to explain (1) The fast lateral growth and to predict its velocity, (2) The slow vertical growth and to predict its velocity, and (3) The uni-modal instead of the bi-modal grain size distribution.

For a direct comparison, we have prepared both the (111) oriented nt-Cu and the ordinary polycrystalline Cu (without nanotwins) of several microns in thickness by electroplating on Si substrates. We will not report here the bi-modal abnormal grain growth in the ordinary polycrystalline Cu because it is well known.

Furthermore, we have performed kinetic modelling of uni-modal abnormal grain growth in the (111) oriented nt-Cu, in which we attempted to provide an analysis of the highly anisotropic growth rate between the lateral and the vertical directions. We found that the major difference is in mobility across the growing grain boundary.

## Materials and methods

The substrates for the < 111 > -oriented nt-Cu films were pieces of 1 × 3 cm^2^ Si cut from a 12-inch diameter silicon wafer. They were cleaned with citric acid solution to remove native surface oxides, before the sputtering of an adhesion layer of 100 nm Ti and a copper seed layer of 200 nm. The latter is highly < 111 > -oriented. A power supply, Keithley 2400, was monitored by a computer to apply current for electrodeposition. We put a Cu electrode to be the anode in the electroplating tank, and the cleaned substrate to be the cathode. The electroplating bath was high-purity CuSO_4_ solution composed of 0.8 M Cu cations, including 80 ppm chloride. An additive with 4000 ppm provided by Chemleaders INC was added for the growth of (111) nanotwins. The magnet stirring rate was 1200 rpm per minute. The deposition time was 8 min using DC electroplating.

For microstructure analysis of the nt-Cu films, dual-beam focused ion beam (DB-FIB, FEI 200) was used to examine the grain and twin structures. Electron backscatter diffraction (EBSD, JEOL 7800F field emission scanning electron microscope with an Oxford system) and orientation image map (OIM) software was used to analyze the grain orientation of the Cu surfaces and grain sizes of the Cu films.

## Results

### Kinetic analysis of grain growth

**Figure 1:**
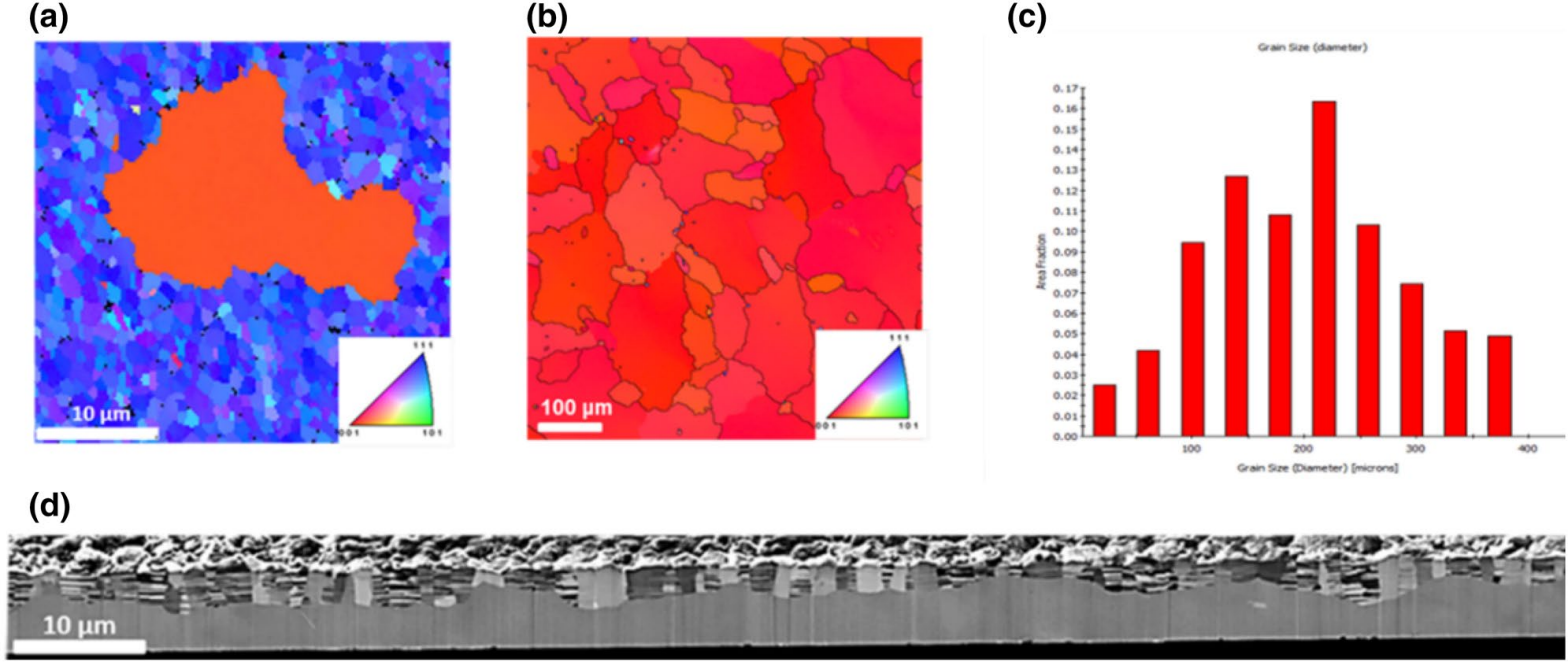
The uni-modal distribution of very large grains in 7 μm thick nt-Cu. (**a**) After annealing at 400 °C for 5 min, a large (200) grain grew in the matrix of small (111) grains. (**b**) Plan-view EBSD showing large (100) grains after 400 °C for 1 h. (**c**) Distribution of grains size in (**b**). (**d**) Cross-sectional FIB image revealing the anisotropic grain growth in the nt-Cu film. A large (100) grain grew at the bottom of the nt-Cu film.

Figure 1 shows the experimental results for the abnormal grain growth. The grain size is 1.5 μm for the as-deposited (111) nt-Cu film of 7 μm thick. A large (100) grain grew and consumed tens of (111) small grains after annealing at 400 °C for 5 min, as seen in Fig. 1a. Upon further annealing at 400 °C for 1 h, the entire (111) nt-Cu were transformed into large (100) grains, as shown in Fig. 1b. In addition, the distribution of grain size is uni-modal, as illustrated in Fig. 1c. The average grain size was 180 μm. The grain growth was highly anisotropic, with a very high rate along the lateral direction, but slowly along the film thickness direction^12^. Figure 1d shows the cross-sectional FIB image for a nt-Cu film annealed at 400 °C for 10 min. A large (100) grain started to grow at the bottom of the nt-Cu film, and it propagated laterally very fast, but the (111) nt-Cu grains near the film surface were still intact. The detailed microstructures of the annealed nt-Cu films have been reported in our most recent studies^17,18^. We found that the grain boundaries were dominantly (100)-oriented as annealed at 400 °C. It initiated from the bottom and latterly consumed the (111) columnar grains by minimizing the twin, surface/interface, and strain energy density. We found that the (100)-oriented grains grew faster at the bottom than at the top of the film. We observed that the (100)-oriented grains accounted for 92% for the total grains in the annealed nt-Cu.

**Figure 2:**
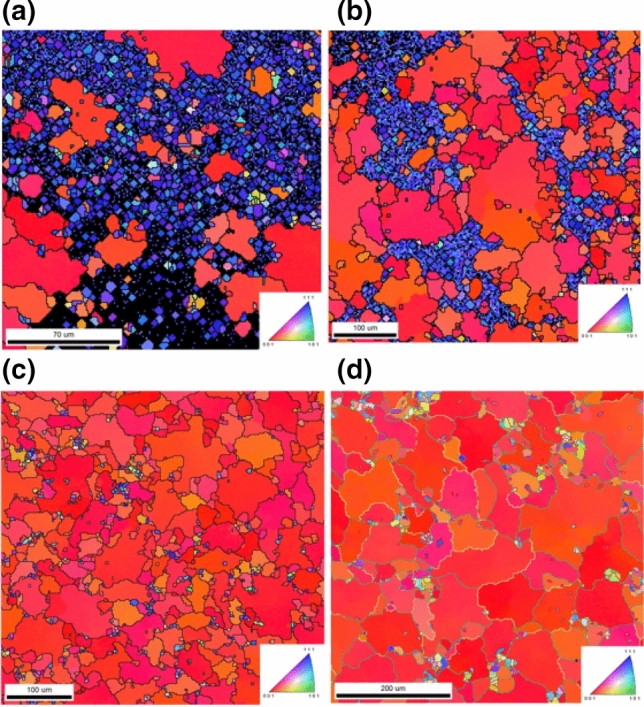
Plan-view EBSD images of the nt-Cu films annealed at various temperatures for 1 h: (**a**) 200 °C; (**b**) 250 °C; (**c**) 300 °C; (**d**) 350 °C.

**Figure 3:**
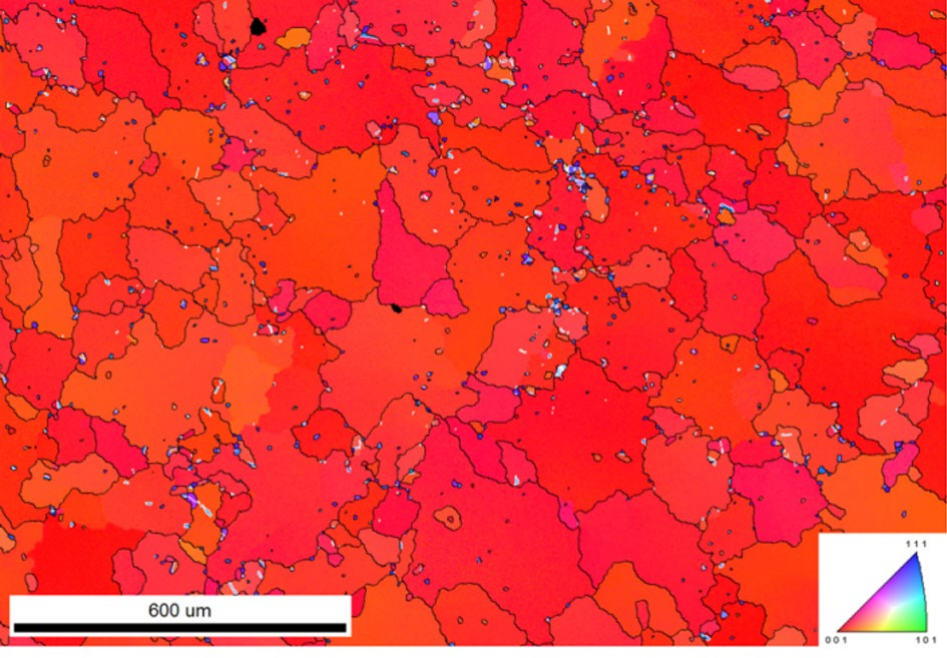
Plan-view EBSD image (inverse pole in *z*-axis) of the nt-Cu annealed at 400 °C for 1 h showing some *Σ*3 grain boundaries (white regions).

We also annealed the nt-Cu at different temperatures, as shown in Fig. 2. It is obvious that the nt-Cu grains gradually grew to large (100) grains. The (111) texture still existed in the films annealed at 250 °C. As annealed at 350 °C, almost Cu grains transformed to the (100) orientation. This could be considered as the critical temperature for orientation transformation. The plan-view EBSD image of the nt-Cu annealed at 400 °C for 1 h with some *Σ*3 grain boundaries (white) is shown in Fig. 3. In fact, the incoherent *Σ*3 grain boundaries and defects coexisted in the nt-Cu affecting the activation energy of breaking the grains prior to the atomic diffusion. Since they were not dominantly distributed in the nt-Cu, their influences could be excluded in our model for the mathematical simplicity.

**Figure 4:**
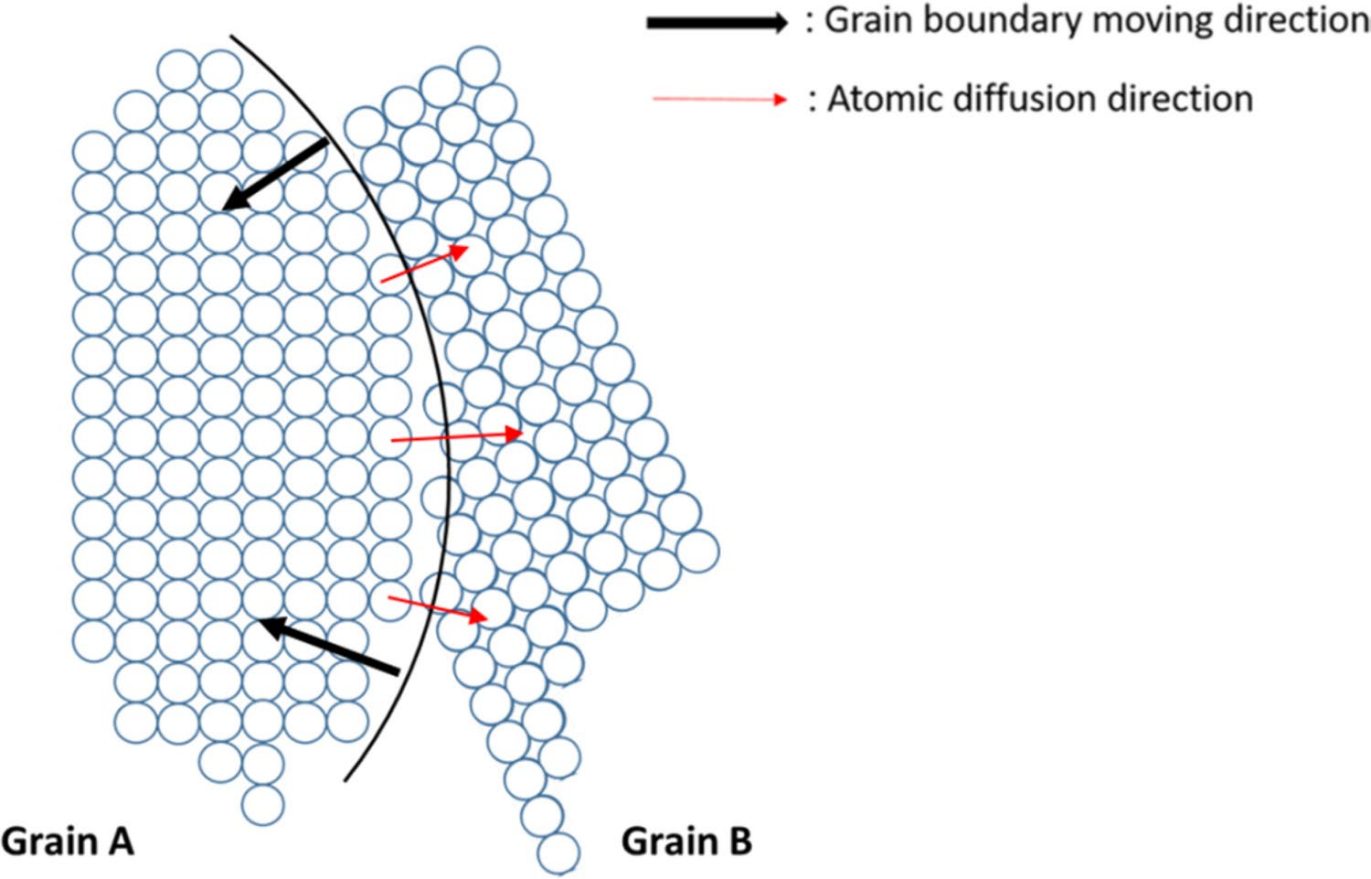
Scheme diagram of grain boundary migration by atomic transition from grain A to grain B.

First, we shall review briefly the mobility and driving force of an ordinary grain growth. Figure 4 shows a schematic diagram of two grains with a curved grain boundary between them. The grain boundary tends to move to the left as indicated by the two black arrows. The movement is the consequence of atoms on the left-hand side of the boundary attempting to jump across the boundary and to take up the positions in the right-hand side grain as indicated by the circles. The driving force and mobility will be given below.

Later, we consider the grain growth in nt-Cu having the uni-directionally (111) oriented microstructure where the highly anisotropic grain growth occurs. We show that the highly anisotropic grain growth is mainly due to the large difference in mobility between the lateral and the vertical growth.

#### Driving force and mobility in grain growth

In Fig. 4, the grain growth is realized via the movement of curved grain boundaries (GBs) driven by Laplace pressure and other effective pressures. Velocity U of the movement is given by Eq. (1) below,

1 $$U\left( \frac{m}{s} \right) = M\left( {\frac{m}{s \cdot Pa}} \right) \cdot P\left( {Pa} \right)$$

where M is mobility and P is effective pressure or driving force. The latter is actually the derivative of Gibbs free energy over the volume change of the new grain with the movement of the GB:

2 $$P\left( {Pa} \right) = \frac{1}{\Omega } \cdot \left( { - \frac{\partial G}{{\partial N^{new} }}} \right)$$

where $\Omega$ is atomic volume, dG is a change of Gibbs free energy of the system when dN^new^ atoms change their “citizenship” from the old grain to the new grain due to the moving GB.

On free energy change, we take μ1 and μ2 to be the chemical potential of atoms in grain 1 (the left-hand side grain) and grain 2 (the right-hand side grain), respectively, and μ1 > μ2 because of Laplace pressure (P = 2γ/r), where r and γ are radius and grain boundary energy, respectively. Thus, in Eq. (2), dG = [(μ1 − μ2)]dN^new^. If the thermodynamic reason of GB migration is related only to the curvature of GB then

$$\mu_{1} - \mu_{2} = P^{Laplace} \Omega = \frac{2\gamma }{r}\Omega$$

The gain of energy by one atom jumping across the grain boundary is $\Delta \mu = \mu_{1} - \mu_{2} = \frac{2\gamma }{r}\Omega$, which is the driving force per atom in grain growth.

Now, we consider a steady state movement of a unit area of the curved grain boundary, the atomic flux equation can be given below.

3 $$J = CU = CMF = C\frac{D}{kT}\left( { - \frac{d\mu }{{dx}}} \right)$$

where U = MF is drift velocity, and M is mobility, F is driving force, and μ is chemical potential. We note that the concentration C = 1/Ω in a pure metal such as Cu. In atomic diffusion, the mobility is determined by diffusivity via Nernst-Einstein equation (D/kT), and force is just the gradient of chemical potential.

On grain growth, the atomic diffusion is across a grain boundary of width of δ, thus

$$U = M^{atomic} \cdot \left( { - \frac{d\mu }{{dx}}} \right) = \frac{{D_{ \bot }^{*} }}{kT} \cdot \left( { - \frac{\Delta \mu }{\delta }} \right) = \frac{{D_{ \bot }^{*} \Omega }}{kT\delta } \cdot \left( { - \frac{\Delta \mu }{\Omega }} \right)$$

Here we have transformed Eq. (3), by taking into account that the ratio of chemical potential change and atomic volume is actually the change of Gibbs–Thomson potential due to transition of the atom from one grain to the opposite grain.

Therefore, we may reformulate the grain boundary velocity as

4 $$U = \frac{{D_{ \bot }^{*} \Omega }}{kT\delta } \cdot \left( { - \frac{dG}{{\Omega dN}}} \right) = \frac{{D_{ \bot }^{*} \Omega }}{kT\delta } \cdot \left( { - \frac{\partial G}{{dV}}} \right) = M^{GB} \cdot P^{effective}$$

where the mobility for the boundary movement during grain growth is

5 $$M = \frac{{D_{ \bot }^{*} \Omega }}{kT\delta }$$

Actually, this relation was suggested by Turnbull^19^. Here $D_{ \bot }^{*}$ is a self-diffusivity “across” the GB. Of course, it is quite different from the diffusivity “along” the GB, $D_{\parallel }^{*}$, which is usually meant in the measurable product of “δD_GB_”, according to Fisher model of grain boundary diffusion^20^. In the following, we consider two ways to modify Eq. (5).

#### To improve Turnbull’s expression for mobility

We note that the transfer of atoms from one grain to the neighboring grain is controlled mainly not by the transversal jump itself but instead by two processes along the two “sides” of the same GB in Fig. 4. Both “walls” (sides) of the grain boundary usually are rather far apart from the ideally flat planar form. Instead, they may be closer to TLK-model (terrace-ledge-kink) of the free crystalline surfaces^21^. At one side there are some preferable places (kinks) for “leaving” the old grain (detachment from grain 1), and at the opposite side there are also some convenient places for “landing” or joining the lattice of the new grain (attachment to grain 2). Since atoms usually should migrate along GB to find suitable step/kink to join the new grain, we should in general, take into account three parts of the transition time:

6 $$\frac{{\delta^{2} }}{{D_{ \bot }^{*} }} = \tau^{\det achment} + \tau^{alongGB} + \tau^{attachment}$$

#### Analysis of the transition times

##### Possibility 1

Time of migration along the GB is much shorter than the time of detachment/attachment.

This possibility was considered in Ref.^22–24^. Omitting some details and rephrasing some arguments in^22^^,^^24^, we may take the usual expression in Eq. (5) and multiply it by the factors $f^{leaving}$ and $f^{parking}$, meaning the fraction of sites suitable for leaving and parking at the sides of GB:

7 $$M^{(1)}_{2 \to 1} = \frac{{D_{ \bot }^{*} \Omega }}{kT\delta }f_{1}^{leaving} f_{2}^{parking}$$

Note that the place which is most convenient for leaving, may not be the most convenient for parking at the same side of GB: $f_{1}^{leaving} \ne f_{1}^{parking} ,\,f_{2}^{leaving} \ne f_{2}^{parking}$(yet, see Eq. (14) below).

In this case the so-called “diffusivity across the GB” is about

8a $$D_{ \bot }^{*} \approx \delta^{2} \nu_{Debye} \exp \left( { - \frac{{\Delta G^{a} }}{kT}} \right),$$

8b $$M^{(1)}_{2 \to 1} = \frac{{\Omega \delta \nu_{Debye} }}{kT}\exp \left( { - \frac{{\Delta G^{a} }}{kT}} \right)f_{1}^{leaving} f_{2}^{parking} .$$

($\Delta G^{a}$ is an activation energy of breaking away from the grain, see^19^)

##### Possibility 2

Time of migration along the GB is much longer than the time of detachment/attachment.

In this case we assume that the time of transition across the GB is equal to the time of migration from the leaving place to the parking place along the GB:

9 $$\frac{{\delta^{2} }}{{D_{ \bot }^{*} }} \approx \frac{{l^{2} }}{{D_{//GB}^{*} }},$$

where $l^{2}$ is a mean square of migration length from “leaving place” to the “parking place” at the neighboring grain wall. Then

10a $$D_{ \bot }^{*} \approx \frac{{\delta^{2} }}{2\tau \bot } = \frac{{\delta^{2} }}{{\left( {l^{2} /D_{//GB}^{*} } \right)}} \approx \frac{{\delta^{2} }}{{l^{2} }}D_{//GB}^{*} ,$$

Thus,

10b $$M^{(2)} = \frac{{\left( {\delta \cdot D_{GB}^{*} } \right)\Omega }}{{2kTl^{2} }}.$$

##### Possibility 3

General case.

Naturally, it contains the previous two cases as the limiting cases. Here we find a more general expression for high angle GB mobility taking into account the finite time of detachment from one grain, migration and looking for parking, and finally, attachment to the new grain. We will respectively consider three fluxes which, in steady-state regime, should be equal to each other:

1. Flux $J_{old \to GB}^{leaving}$ of atoms leaving the old grain,
2. Flux $J_{along\,GB}^{migrating}$ of atoms which had already left old grain but are still migrating along the GB and looking for parking place,
3. Flux $J_{GB \to new}^{parking}$ of atoms parking to the new grain.
  11 $$J_{old \to GB}^{leaving} = J_{along\,GB}^{migrating} = J_{GB \to new}^{parking} \equiv J = \frac{A}{\Omega } \cdot V_{GB}$$

Here $V_{GB}$ is a GB velocity,$A$ is a total area of GB, $\Omega$ is an atomic volume.

First and third fluxes are the differences of two opposite fluxes

12, leaving $$J_{old \to GB}^{leaving} = \frac{A}{{A_{1old} }}f_{old}^{leaving} \nu_{D} \exp \left( { - \frac{{E_{old/GB}^{S} - E_{old} }}{kT}} \right) - A\delta \cdot n_{GB/old}^{mobile} f_{old}^{parking} \nu^{\prime}_{D} \exp \left( { - \frac{{E_{old/GB}^{S} - E_{GB} }}{kT}} \right)$$

12, parking $$J_{GB \to new}^{parking} = - \frac{A}{{A_{1new} }}f_{new}^{leaving} \nu_{D} \exp \left( { - \frac{{E_{new/GB}^{S} - E_{new} }}{kT}} \right) + A\delta \cdot n_{GB/new}^{mobile} f_{new}^{parking} \nu^{\prime}_{D} \exp \left( { - \frac{{E_{new/GB}^{S} - E_{GB} }}{kT}} \right)$$

12, migrating $$J_{along\,GB}^{migrating} = A \cdot D_{ \bot }^{ef} \frac{{n_{GB/old}^{mobile} - n_{GB/new}^{mobile} }}{\delta }.$$

Here $A_{1new}$ and $A_{1old}$ are the areas per atom at the new grain-wall of GB and old grain-wall of GB, respectively, $\nu_{D}$ and $\nu^{\prime}_{D}$ are the Debye frequencies for the atoms belonging to the grain and to the GB (migrating atom), respectively, $E_{old/GB}^{S}$ and $E_{new/GB}^{S}$ are the saddle-point energy of the barriers, separating old or new grain from GB-sublayer. $n_{GB/old}^{mobile}$ and $n_{GB/new}^{mobile}$ are the average concentrations of migrating atoms near the leaving places of the old grain and near the parking places of the new grain.

Substituting Eqs. (12) into Eqs. (11), we obtain two equations for two unknowns, $n_{GB/old}^{mobile}$ and $n_{GB/new}^{mobile}$. In the following we consider the energy difference between two grains to be rather small and use the simple expansion:

$$\Delta E \equiv E_{old} - E_{new} ,\,\,\overline{E} = \left( {E_{old} + E_{new} } \right)/2$$

$$\begin{aligned} & \exp \left( {\frac{{E_{old} - E_{new} }}{2kT}} \right) \equiv \exp \left( {\frac{\Delta E}{{2kT}}} \right) = \cosh \left( {\frac{\Delta E}{{2kT}}} \right) + \sinh \left( {\frac{\Delta E}{{2kT}}} \right) \approx 1 + \frac{\Delta E}{{2kT}} \\ & \exp \left( {\frac{{E_{new} - E_{old} }}{2kT}} \right) \equiv \exp \left( { - \frac{\Delta E}{{2kT}}} \right) = \cosh \left( {\frac{\Delta E}{{2kT}}} \right) - \sinh \left( {\frac{\Delta E}{{2kT}}} \right) \approx 1 - \frac{\Delta E}{{2kT}} \\ \end{aligned}$$

Then a simple algebra gives:

13 $$\begin{aligned} & V_{GB} = \Omega \cdot D_{ \bot }^{ef} \frac{{n_{GB/old}^{mobile} - n_{GB/new}^{mobile} }}{\delta } \\ & \quad = \Omega \frac{{\nu_{D} \left( {\left( {\frac{{f_{old}^{leaving} f_{new}^{parking} }}{{A_{1old} }} - \frac{{f_{new}^{leaving} f_{old}^{parking} }}{{A_{1new} }}} \right)\cosh \left( {\frac{\Delta E}{{2kT}}} \right) + \left( {\frac{{f_{old}^{leaving} f_{new}^{parking} }}{{A_{1old} }} + \frac{{f_{new}^{leaving} f_{old}^{parking} }}{{A_{1new} }}} \right)sinh\left( {\frac{\Delta E}{{2kT}}} \right)} \right)}}{{f_{new}^{parking} \exp \left( {\frac{{E_{old/GB}^{S} - E_{GB} }}{kT}} \right) + f_{old}^{leaving} \exp \left( {\frac{{E_{new/GB}^{S} - E_{GB} }}{kT}} \right) + \frac{{\delta^{2} f_{old}^{leaving} f_{new}^{parking} \nu^{\prime}_{D} }}{{D_{ \bot }^{ef} }}}} \\ \end{aligned}$$

It seems obvious that at $\Delta E = 0$ the GB velocity should be zero. (No driving force – no movement). For this, the following condition should be provided:

14 $$\frac{{f_{old}^{leaving} f_{new}^{parking} }}{{A_{1old} }} = \frac{{f_{new}^{leaving} f_{old}^{parking} }}{{A_{1new} }}$$

Under this condition (14) and under approximations $\frac{\Delta E}{{2kT}} \ll 1 \Rightarrow sinh\left( {\frac{\Delta E}{{2kT}}} \right) \approx \frac{\Delta E}{{2kT}}$, $\nu^{\prime}_{D} \approx \nu_{D}$ one gets:

15a $$\begin{aligned} & V_{GB} = \Omega \cdot D_{ \bot }^{ef} \frac{{n_{GB/old}^{mobile} - n_{GB/new}^{mobile} }}{\delta } \\ & \quad = \frac{\Omega }{{A_{1old} }}\frac{{f_{old}^{leaving} f_{new}^{parking} \nu_{D} }}{{f_{new}^{parking} \exp \left( {\frac{{E_{old/GB}^{S} - E_{GB} }}{kT}} \right) + f_{old}^{leaving} \exp \left( {\frac{{E_{new/GB}^{S} - E_{GB} }}{kT}} \right) + \frac{{\delta^{2} f_{old}^{leaving} f_{new}^{parking} \nu_{D} }}{{D_{ \bot }^{ef} }}}}\frac{\Delta E}{{kT}} \\ \end{aligned}$$

Or, in other form:

15b $$V_{GB} = \frac{\Omega }{{A_{1old} }}\frac{{\frac{{D_{ \bot }^{ef} }}{{\delta^{2} }}}}{{1 + \frac{{D_{ \bot }^{ef} }}{{\delta^{2} \nu_{D} }}\left( {\exp \left( {\frac{{E_{old/GB}^{S} - E_{GB} }}{kT}} \right)/f_{old}^{leaving} + \exp \left( {\frac{{E_{new/GB}^{S} - E_{GB} }}{kT}} \right)/f_{new}^{parking} } \right)}}\frac{{E_{old} - E_{new} }}{kT}$$

Note that, as before in Eq. (9), we suggest $\frac{{D_{ \bot }^{*} }}{{\delta^{2} }} \approx \frac{{D_{//GB}^{*} }}{{l^{2} }}$ with $l$ being the mean free path length of migration along GB before finding the parking place.

### Mobility of horizontal boundary versus vertical boundary between a (200) grain and the columnar nanotwinned grains

**Figure 5:**
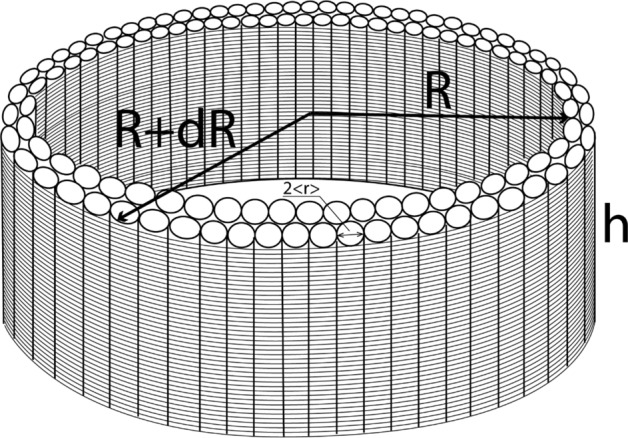
Abnormal growth of the large cylintrical 200-grain at the expense of the surrounding nanotwinned columnar grains.

We consider the abnormal growth of a new (200) grain in the columnar nanotwinned grains, as shown in Fig. 5. The shape of the (200) grain is a circular disc of radius R and height H.

We will analyze the driving force and mobility separately for the lateral growth (when R grows and H is constant) and the vertical growth (when H grows and R is constant). If the latter is much slower than the former, it is uni-modal of abnormal growth.

#### Time of migration along the GB is much shorter than the time of detachment/attachment

**Figure 6:**
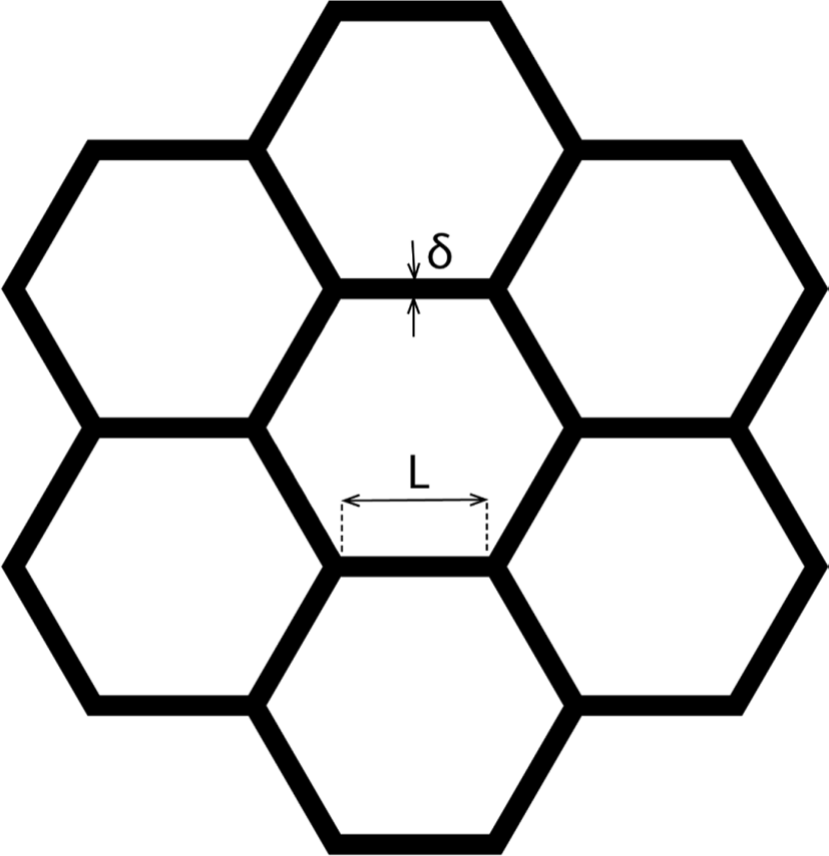
Idealized plan-view of the columnar structure of the nanotwinned grains (black thick lines—are the triple junctions which are good leaving/parking places for atoms crossing the interface with the 200-grain).

Consider a horizontal interfacial boundary plane between the top side of a large (200) grain and the bottom side of a set of small columnar nanotwinned grains, each of which has a horizontal area about L * L = (2.3 μm) × (2.3 μm), where 2.3 μm is the average grain size of the columnar hexagonal grains, see Fig. 6. For the convenience of calculation, we assume a hexagonal grain size having triple junctions among the columnar grains.

The most suitable places for leaving or landing of atoms are those horizontal triple junctions of the big 200-grain and two columnar grains consisting of lines of length Land thickness $\delta$. Thus, the fraction of suitable parking and leaving places is (in case of hexagonal lattice),

16 $$f_{\parallel }^{leaving\,nanotw} \sim \frac{6/2 \cdot \delta \cdot l}{{6 \cdot \frac{1}{2}L^{2} \frac{\sqrt 3 }{2}}} = \frac{2\delta }{{\sqrt 3 L}} \approx \frac{\delta }{L}$$

**Figure 7:**
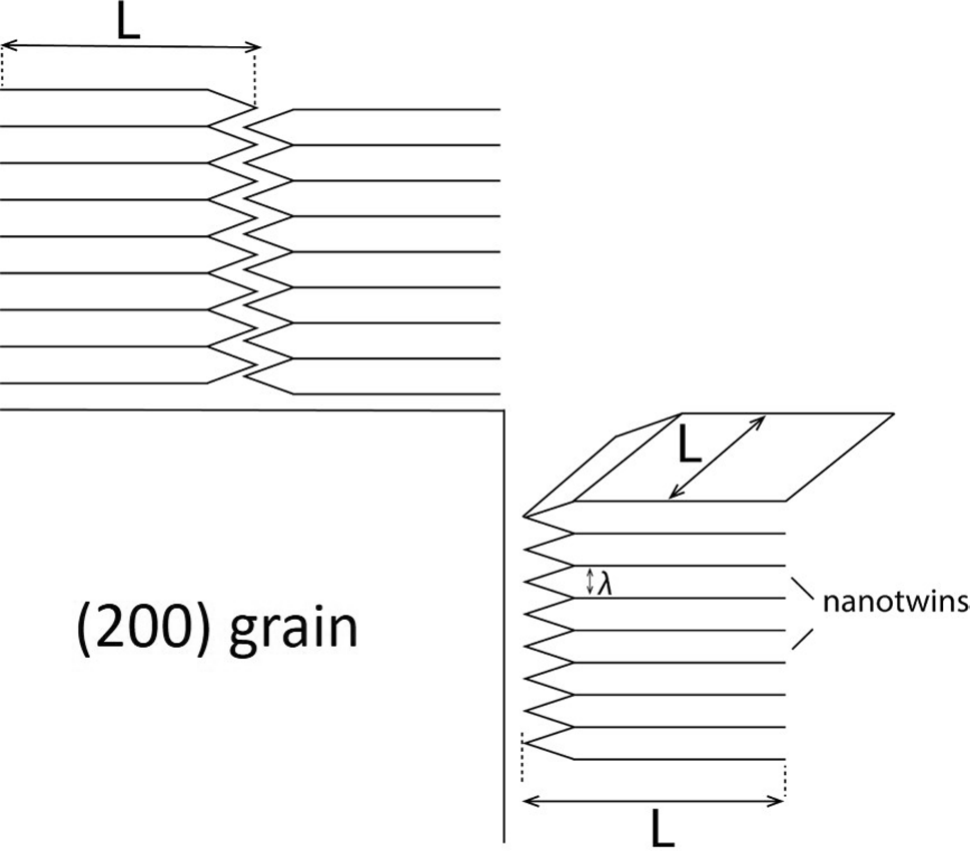
Difference of leaving/parking places at the “vertical” and “horizontal” boundaries of the 200 grain.

Next, we consider the cross-section of a vertical interfacial boundary plane between the (200) grain and columnar nanotwinned grains with vertical facets of length *L*, thickness $\delta$ and twin spacing $\lambda$, see Fig. 7.

In this case the fraction of suitable parking and leaving places on the vertical interface

17 $$f_{ \bot }^{leaving\,nanotw} \sim \frac{\delta \cdot L}{{\lambda L}} = \frac{\delta }{\lambda }$$

The parking abilities for two different facets of the large 200- grain are, in general, different but of the same order of magnitude, and we will take them to be the same: $f_{\parallel }^{parking\,200} \approx f_{ \bot }^{parking\,200}$.

Thus, in case 1 of Turnbull modification, we obtain:

18 $$\frac{{M_{ \bot }^{(1)} }}{{M_{\parallel }^{(1)} }} = \frac{{f_{ \bot }^{leaving\,nanotw} }}{{f_{\parallel }^{leaving\,nanotw} }} \approx \frac{L}{\lambda } = \left( {\frac{2300}{{40}}} \right) \approx 60$$

#### Time of migration along the GB is much longer than the time of detachment/attachment

According to Eq. (13),

19 $$\frac{{M_{ \bot }^{(2)} }}{{M_{\parallel }^{(2)} }} \approx \frac{{l_{\parallel }^{2} }}{{l_{ \bot }^{2} }}.$$

Geometrically, for “horizontal” grain boundary between abnormal grain 200 and nanotwinned grains, $l_{\parallel } \sim L$. For “vertical” grain boundary between abnormal grain 200 and nanotwinned grains, $l_{ \bot } \sim \lambda$. Thus,

20 $$\frac{{M_{ \bot }^{(1)} }}{{M_{\parallel }^{(1)} }} \approx \left( {\frac{L}{\lambda }} \right)^{2} = \left( {\frac{2300}{{40}}} \right)^{2} \approx 3300$$

Thus, we have obtained the reason for the highly anisotropic abnormal growth. Both results from Eqs. (18) and (20) for two different theoretical models (case 1 and case 2) differ significantly, but both of them explain a very fast horizontal abnormal growth.

### Alternative (thermodynamic) possibility of the fast horizontal growth—shape phase transition from a (200) oriented nucleus

In the previous section we analyzed the possible kinetic reasons of very strong growth anisotropy, related to anisotropy of GB mobility for GB with nanotwinned grains. Below we will consider the alternative of thermodynamic (energetic) possibility.

On nucleation of the (200) nucleus, it will have the following Gibbs free energy changes, in forming a disc with a radius of R and thickness of H. All the changes are denoted as a Gibbs free energy gain. First term is a gain in bulk free energy per atom ($\Delta g^{bulk}$) multiplied by the number of atoms in the (200)-grain. This energy gain is due to elimination of extra thermal stress and defects in the bulk per atom:

21 $$\Delta G_{1} = \Delta g^{bulk} \frac{{\pi R^{2} H}}{\Omega },\,\Delta g^{bulk} \equiv \left( {g_{new}^{bulk} - g_{old}^{bulk} } \right) < 0$$

The growth of this new (200) nucleus eliminates the vertical grain boundaries between the columnar old grains. The number of such grains eliminated by the new (200) large cylindrical grain, corresponds to a change of the Gibbs free energy of $\frac{{\pi R^{2} H}}{{\pi \overline{r}^{2} H}}$, where $\overline{r}$ is an average lateral grain size of the old columnar grains.

Each columnar (approximately—cylindrical) grain was responsible for the grain-boundary area of $A_{1} = \frac{1}{2}2\pi rH$.

Thus, the corresponding second term in Gibbs free energy change is

22 $$\Delta G_{2} = - \frac{{R^{2} }}{{\overline{r}^{2} }}\gamma \frac{1}{2}2\pi \overline{r}H = - \gamma \pi \frac{{R^{2} H}}{{\overline{r}}}$$

Furthermore, we have additional side areas of cylinder which are of the “ceiling” of the (200) grain, so that

23 $$\Delta G_{3} = + \gamma^{new/old} 2\pi RH + \gamma^{new/old} \pi R^{2}$$

And the last is the change of energy at the bottom side. It includes two terms (both proportional to the area $\pi R^{2}$). First term is the change of surface energy between the (200) grain and the Si substrate and the seed layer. Second term is an energetic profit due to transformation of all seed layer or part of it into the new 200-grain:

24 $$\Delta G_{4} = \left( {\gamma^{new/seed} - \gamma^{old/seed} } \right)\pi R^{2} + \left( { - \Delta g_{seed \to 200} } \right)\frac{{\pi R^{2} \delta_{seed \to 200} }}{\Omega }$$

Here $\Delta g_{seed \to 200}$ is an energetic profit (per one atom) of transformation of the highly defect-rich seed layer into 200-grain, $\delta_{seed \to 200}$ is a thickness of transformed seed layer.

In total, we obtain,

25 $$\Delta G_{total} = \Delta G_{1} + \Delta G_{2} + \Delta G_{3} + \Delta G_{4} = - aR^{2} H + bRH + cR^{2}$$

Here a is always positive (minus sign is written explicitly), b is also always positive, but c may be either positive or negative:

26 $$\begin{aligned} & a = \pi \left( {\frac{{\left( { - \Delta g^{bulk} } \right)}}{\Omega } + \frac{\gamma }{{\overline{r}}}} \right)\pi > 0 \\ & b = + 2\pi \gamma^{new/old} > 0 \\ & c = \pi \left( {\gamma^{new/old} + \gamma^{new/seed} - \frac{{\Delta g_{seed \to 200} \delta_{seed \to 200} }}{\Omega } - \gamma^{old/seed} } \right) > 0\,or\, < 0 \\ \end{aligned}$$

We will analyze both cases: c > 0 and c < 0.

Instead of R and H, we introduce volume and shape parameter below;

$$V \equiv \pi R^{2} H,\,\,\,\varphi \equiv H/R,$$

So that, $$R = V^{1/3} \varphi^{ - 1/3} ,H = V^{1/3} \varphi^{2/3}$$.

Thus, in terms of volume and shape parameters

27 $$\Delta G_{total} \left( {V,\varphi } \right) = - aV^{1} + V^{2/3} \left( {b\varphi^{1/3} + c\varphi^{ - 2/3} } \right)$$

It is usual to optimize shape at any given volume, which mathematically means to minimize Gibbs free energy with respect to shape parameter at any given volume:

$$\begin{aligned} & \frac{\partial }{\partial \varphi }\Delta G_{total} \left( {V,\varphi } \right) = 0,\,\,\frac{{\partial^{2} }}{{\partial \varphi^{2} }}\Delta G_{total} \left( {V,\varphi } \right) > 0 \\ & \frac{\partial }{\partial \varphi }\Delta G_{total} \left( {V,\varphi } \right) = V^{2/3} \cdot \frac{1}{3}\left( {b\varphi^{ - 2/3} - 2c\varphi^{ - 5/3} } \right) \\ \end{aligned}$$

The above results are totally different at c > 0 or c < 0, because the shape dependence is very different for the positive and negative sign of c:

$$y = x \pm \frac{\left| c \right|}{b}\frac{1}{{x^{2} }},\,\,where\,\,\,x \equiv \varphi^{1/3}$$

Namely, at c > 0 ($\gamma^{new/old} + \gamma^{new/seed} > \gamma^{old/seed}$) the optimal shape parameter corresponds to the local minimum of

28 $$\varphi^{optimal} = 2\frac{c}{b} = \frac{{\gamma^{new/old} + \gamma^{new/seed} - \frac{{\Delta g_{seed \to 200} \delta_{seed \to 200} }}{\Omega } - \gamma^{old/seed} }}{{\gamma^{new/old} }}$$

In case of c < 0, that is,

29 $$\gamma^{new/old} + \gamma^{new/seed} < \frac{{\Delta g_{seed \to 200} \delta_{seed \to 200} }}{\Omega } + \gamma^{old/seed}$$

the dependence has no minimum, so that we have “shape phase transition”, meaning the transformation of the (200) cylinder grain into an infinitely wide disc. Physically it means that the nuclei should grow only laterally—theoretically infinitely, and practically—until it meets another new grain.

This may explain mathematically why the new grains first grow only laterally, and then, slowly—vertically. Vertical growth starts after filling all surfaces with big new grains. This can also explain the mono-size distribution: if the nucleation time is not very small, and lateral growth time is very small, the small grains will have no time to nucleate, thus the respective places will be occupied by the very first grains. Numeric analysis of the possible realization of condition (29) will be made elsewhere.

## Discussions

Both reasons of kinetic (anisotropy of grain boundary mobility depending on nanotwins configuration) as well as thermodynamic (energetic favor of elongation in horizontal directions) act synergetically and lead to extremely fast and extremely anisotropic abnormal 200-grain growth at the expense of pre-existing nanotwinned structure. A very important question is whether the joints between nanotwin boundaries and grain boundary (with 200-grain) are indeed the convenient places for leaving or parking. In general, our experimental conditions show that the nanotwins boundaries may not be fully coherent and they are semi-coherent, also they contain effective vacancy sinks causing the elimination of Kirkendall voids in reactions between nanotwinned copper and tin, as reported in Ref.^25^.

Figure 1a shows a bimodal distribution, which is a transient phenomenon in the beginning of the transformation, and it will quickly disappear and become uni-model, as shown in Fig. 1b. In Fig. 1d, the cross-sectional image shows that the interface between the extremely large (100) oriented grain at the bottom and the tiny (111) oriented nt-Cu grains on top are not uniform. It means the vertical growth rate is not constant across the entire interface, so certain parts could grow slightly faster, which can lead to the transient bi-model distribution as shown in Fig. 1a. On the other hand, whether we can achieve a uniform vertical growth rate is challenging.

Morphologically, the vertical growth interface is parallel to the (111) twin plane in the nt-Cu. This is the key reason of the finding of uni-model growth, because on the (111) plane, while there are lattice sites in terrace-ledge-kink, which can serve as the leaving sites in the kinetic process, they are few and slow! Therefore, an interesting question to consider is if we use randomly oriented nt-Cu, instead of (111) oriented nt-Cu, we might not have the uni-model growth. This could be a study in the future.

In the last Section, even though we consider the thermodynamic reason for the anisotropic growth. However, in kinetics, it is the slowest process which dominates the outcome. Thus, we conclude below that it is mainly due to the difference in mobility between the vertical growth and the lateral growth, which leads to the highly anisotropic growth.

## Conclusions

Grain growth velocity, U = MF, can be controlled by the mobility M, or the driving force F, or both. For (111) oriented nt-Cu films, because of the highly anisotropic microstructure, our kinetic analysis and calculation in the above showed that it is the mobility which dominates the highly anisotropic growth, in which the lateral growth rate can be two orders of magnitude higher than the vertical growth rate. As a consequence, the abnormal grain growth has been converted from bi-modal to uni-modal.

## References

[1] Murarka SP (1997). Multilevel interconnections for ULSI and GSI era. Mater. Sci. Eng. R Rep..

[2] Li B (2014). Electromigration challenges for advanced on-chip Cu interconnects. Microelectron. Reliab..

[3] Li B (2004). Reliability challenges for copper interconnects. Microelectron. Reliab..

[4] Tseng IH (2021). Electromigration failure mechanisms of <111>-oriented nanotwinned Cu redistribution lines with polyimide capping. Results Phys..

[5] Li YJ (2020). Tensile properties of <111>-oriented nanotwinned Cu with different columnar grain structures. Materials.

[6] Cheng HY (2021). Effect of deposition temperature on mechanical properties of nanotwinned Cu fabricated by rotary electroplating. Mater. Sci. Eng. A.

[7] Tran DP (2021). Electrodeposition of slanted nanotwinned Cu foils with high strength and ductility. Electrochim. Acta.

[8] Hung Y-W (2021). Effect of Cu ion concentration on microstructures and mechanical properties of nanotwinned Cu foils fabricated by rotary electroplating. Nanomaterials.

[9] Lu L (2004). Ultrahigh strength and high electrical conductivity in copper. Science.

[10] Lu L, Chen X, Huang X, Lu K (2009). Revealing the maximum strength in nanotwinned copper. Science.

[11] Hsiao HY (2012). Unidirectional growth of microbumps on (111)-oriented and nanotwinned copper. Science.

[12] Liu TC (2012). Fabrication and characterization of (111)-oriented and nanotwinned Cu by DC electrodeposition. Cryst. Growth Des..

[13] Lu CL (2014). Extremely anisotropic single-crystal growth in nanotwinned copper. NPG Asia Mater..

[14] Xu D (2007). Nanotwin formation in copper thin films by stress/strain relaxation in pulse electrodeposition. Appl. Phys. Lett..

[15] Thompson CV, Carel R (1996). Stress and grain growth in thin films. J. Mech. Phys. Solids.

[16] Sonnweber-Ribic P (2012). Kinetics and driving forces of abnormal grain growth in thin Cu films. Acta Mater..

[17] Tseng C-H (2020). Kinetic study of grain growth in highly (111)-preferred nanotwinned copper films. Mater. Charact..

[18] Tseng I-H (2021). Effect of thermal stress on anisotropic grain growth in nano-twinned and un-twinned copper films. Acta Mater..

[19] Turnbull D (1951). Theory of grain boundary migration rates. JOM.

[20] Surholt T, Herzig C (1997). Grain boundary self-diffusion in Cu polycrystals of different purity. Acta Mater..

[21] Stranski IN (1928). Zur Theorie des Kristallwachstums. Z. Phys. Chem..

[22] Porter DA, Easterling KE, Sherif M (2009). Phase transformations in metals and alloys (Revised Reprint).

[23] Rollett AD, Gottstein G, Shvindlerman LS, Molodov DA (2004). Grain boundary mobility–a brief review. Z. Metallkd..

[24] Gottstein G, Shvindlerman LS (2009). Grain boundary migration in metals: Thermodynamics, kinetics, applications.

[25] Liu TC (2013). Eliminate Kirkendall voids in solder reactions on nanotwinned copper. Scr. Mater..

